# Senescent cell transplantation into the skin induces age‐related peripheral dysfunction and cognitive decline

**DOI:** 10.1111/acel.14340

**Published:** 2024-10-07

**Authors:** Ana Catarina Franco, Helene Martini, Stella Victorelli, Anthony B. Lagnado, Saranya P. Wyles, Jennifer L. Rowsey, Nicholas Pirius, Seung‐Hwa Woo, Daniela G. Costa, Selim Chaib, Stefan G. Tullius, Tamar Tchkonia, James L. Kirkland, Sundeep Khosla, Diana Jurk, Claudia Cavadas, João F. Passos

**Affiliations:** ^1^ Department of Physiology and Biomedical Engineering Mayo Clinic Rochester Minnesota USA; ^2^ Robert and Arlene Kogod Center on Aging Mayo Clinic Rochester Minnesota USA; ^3^ CNC–Center for Neuroscience and Cell Biology (CNC‐UC) University of Coimbra Coimbra Portugal; ^4^ Centre for Innovation in Biomedicine and Biotechnology (CIBB) University of Coimbra Coimbra Portugal; ^5^ Faculty of Pharmacy University of Coimbra Coimbra Portugal; ^6^ Department of Dermatology Mayo Clinic Rochester Minnesota USA; ^7^ Center for Advanced Gerotherapeutics, Division of Endocrinology Diabetes & Metabolism, Cedars‐Sinai Medical Center Los Angeles CA USA; ^8^ Division of Transplant Surgery, Department of Surgery Harvard Medical School, Brigham and Women's Hospital Boston Massachusetts USA; ^9^ Department of Neurology Mayo Clinic, Rochester Minnesota USA

**Keywords:** cellular senescence, cognitive decline, paracrine senescence, skin aging

## Abstract

Cellular senescence is an established cause of cell and tissue aging. Senescent cells have been shown to increase in multiple organs during aging, including the skin. Here we hypothesized that senescent cells residing in the skin can spread senescence to distant organs, thereby accelerating systemic aging processes. To explore this hypothesis, we initially observed an increase in several markers of senescence in the skin of aging mice. Subsequently, we conducted experiments wherein senescent fibroblasts were transplanted into the dermis of young mice and assessed various age‐associated parameters. Our findings reveal that the presence of senescent cells in the dermal layer of young mice leads to increased senescence in both proximal and distal host tissues, alongside increased frailty, and impaired musculoskeletal function. Additionally, there was a significant decline in cognitive function, concomitant with increased expression of senescence‐associated markers within the hippocampus brain area. These results support the concept that the accumulation of senescent cells in the skin can exert remote effects on other organs including the brain, potentially explaining links between skin and brain disorders and diseases and, contributing to physical and cognitive decline associated with aging.

## Graphical abstract

This study demonstrates that transplanting senescent cells into the skin of young mice accelerates physical and cognitive decline, potentially due to the spread of senescent cells. These findings indicate a cross‐organ communication between the skin and systemic aging processes.

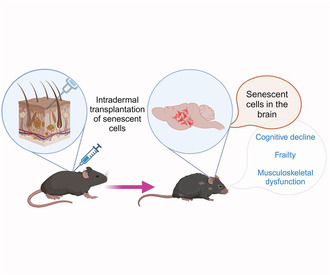

**Abbreviations**
3D: Three‐Dimensional
ANOVA: Analysis of Variance
BSA: Bovine Serum Albumin
CA3: Cornu Ammonis 3
CD: Cluster of Differentiation
CNS: Central Nervous System
CSA: Cross sectional area
DAPI: 4’,6‐Diamidino‐2‐Phenylindole
DMEM: Dulbecco's Modified Eagle Medium
EPM: Elevated Plus Maze
FFPE: Formalin‐fixed paraffin‐embedded
G‐CSF: Granulocyte Colony‐Stimulating Factor
HEPA: High‐Efficiency Particulate Air
HMGB1: High Mobility Group Box 1
IL: Interleukin
LUC: Luciferase
MAFs: Mouse Adult Fibroblast
mRNA: Messenger Ribonucleic Acid
NGS: Normal Goat serum
PBS: Phosphate‐Buffered Salin
PCNA: Proliferating Cell Nuclear Antigen
PFA: paraformaldehyde
Q‐PCR: Quantitative real‐time Polymerase Chain Reaction
RNA‐ISH: RNA In Situ Hybridization
SASP: Senescence‐Associated Secretory Phenotype
SEM: Standard Error of the Mean
TAF: Telomere Associated Foci
UV: Ultraviolet
WGA: Wheat Germ Agglutinin
μCT: Micro‐Computed Tomography

## 1 INTRODUCTION

Cellular senescence is a cell fate triggered by various stresses and characterized by an irreversible growth arrest and a Senescence‐Associated Secretory Phenotype (SASP) (Gorgoulis et al., 2019).

While cellular senescence and the SASP can have beneficial roles in tumor suppression (Serrano et al., 1997), embryonic development (Muñoz‐Espín et al., 2013), and tissue repair (Demaria et al., 2014), chronic SASP has been suggested to contribute to tissue aging and age‐related diseases (Jurk et al., 2014).

Senescent cell accumulation in multiple tissues during aging and age‐related diseases is well‐documented. Studies in mice indicate that removing these cells can improve age‐related conditions, indicating that senescence plays a causal role in age‐related tissue dysfunction (Baker et al., 2016; Xu et al., 2015). However, fundamental questions about how senescent cells arise during aging, their kinetics in different organs and how they propagate in different tissues require further investigation.

Research indicates that senescent cells can induce paracrine senescence in neighboring cells either through soluble SASP factors or via extracellular vesicles (Acosta et al., 2013; Borghesan et al., 2019). Additionally, soluble factors released by senescent cells in one tissue can spread senescence to distant tissues, thereby contributing to systemic effects (Farr et al., 2023; Xu et al., 2018).

The skin, being the largest organ in the body, plays a critical role as a barrier against pathogens, chemicals, and UV radiation. During aging, the skin exhibits various structural, cellular, and molecular alterations, including an accumulation of senescent cells which can be influenced by both intrinsic and extrinsic factors (Victorelli et al., 2019). Interestingly, various studies propose skin aging as a potential predictor for age‐related dysfunction in other organs. Some research has revealed correlations between skin aging and facial appearance with respect to longevity, disease susceptibility, and mortality rates (Gunn et al., 2013, 2016). Additionally, studies have indicated that exposure to ultraviolet radiation in mice may induce impaired hippocampal neurogenesis (Han et al., 2017). This implies the intriguing concept that changes occurring in the skin may potentially manifest as alterations in distal organs, notably including the brain. These observations have led to the yet untested hypothesis that the accumulation of senescent cells within the skin might have the potential to spread senescence to other organs (Franco et al., 2022).

## 2 RESULTS

### 2.1 Markers of cellular senescence increase in aging murine skin

**FIGURE 1:**
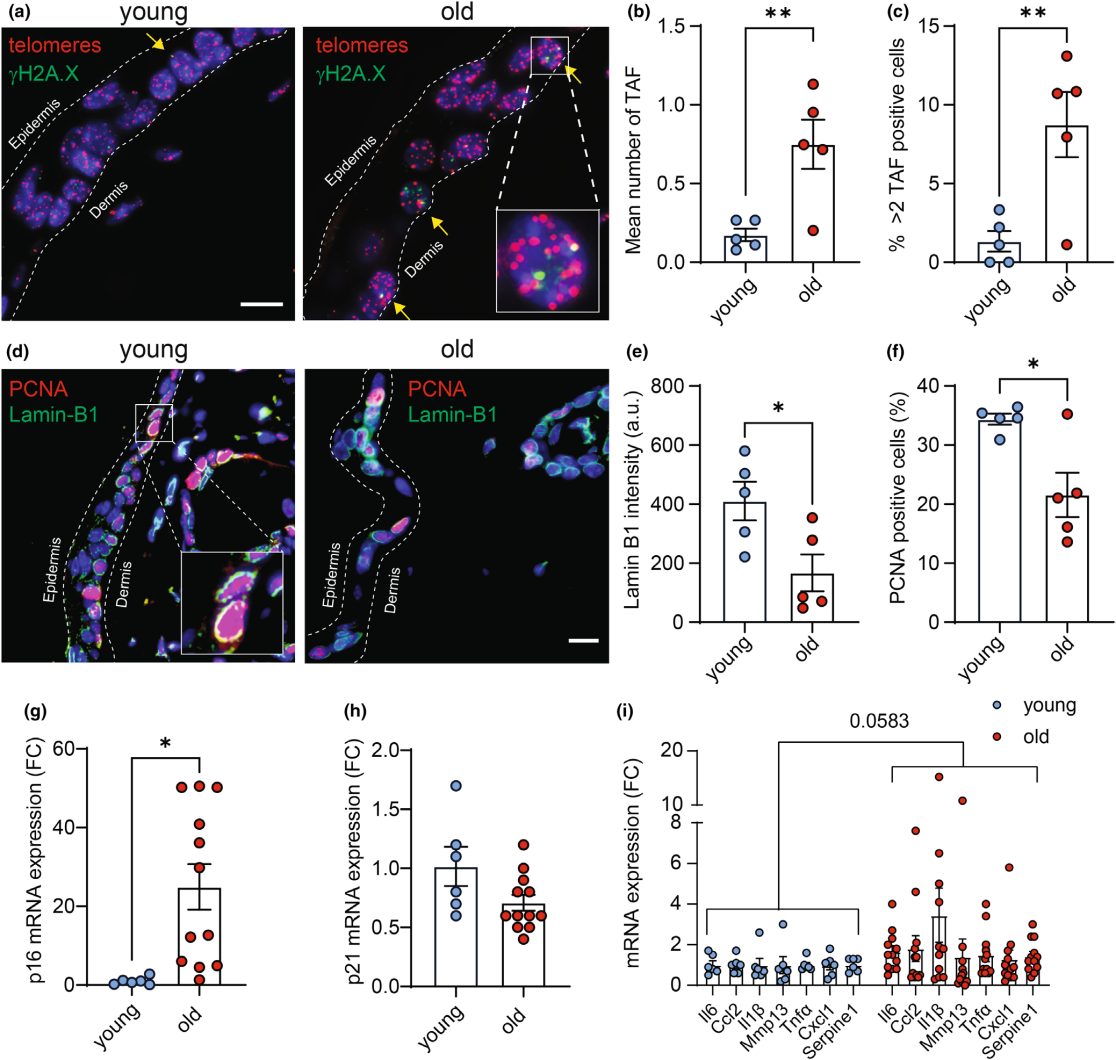
Senescence‐associated markers increase in mouse skin with age. (a) Representative Immuno‐FISH images (using telomere specific nucleic acid probe (CCCTAA) in red and anti‐γH2A.X antibody in green). Arrows indicate co‐localization between telomeres and γH2A.X. (Scale bar 10 μm); (b) Mean number of TAF in skin epidermal layer and (c) percentage of cells presenting more than two TAFs in the epidermis. (d) Representative microscopy images of PCNA (in red) and Lamin‐B1 (in green). (Scale bar 10 μm). (e) Quantification of Lamin‐B1 immunoreactivity and (f) percentage of PCNA positive cells in the epidermis. Data is presented and mean ± S.E.M of 5 animals per group. (g) mRNA levels of p16INK4a; (h) p21 and (i) SASP factors in young and old skin of 6–12 animals. Data are mean ± S.E.M. Statistical significance (*p < 0.05, **p < 0.01) was assessed using Student's *t*‐test. a.u. Arbitrary units.

To first confirm that cellular senescence increases in the skin with age, we analyzed young and old mouse skin for a range of senescence‐associated markers. Telomere‐associated DNA damage is recognized as a hallmark of cellular senescence (Hewitt et al., 2012). We observed a significant increase in the average number of telomere‐associated foci (TAF) *per* cell (Figure 1a,b), as well as an increased the percentage of cells exhibiting more than two TAFs *per* nucleus (Figure 1c) in the epidermis of aged mice compared to young controls. To further evaluate cellular senescence in mouse skin with age, we performed immunofluorescence for Lamin B1, which has been shown to be lost from the nuclear lamina in senescent cells (Freund et al., 2012) and PCNA, a marker of cellular proliferation. We observed a noteworthy decrease in the levels of Lamin B1 (Figure 1d,e) and PCNA (Figure 1d,f) in the skin of aged mice. Subsequently we investigated mRNA levels of the p21 and p16^Ink4a^ (p16) and several SASP components in whole‐skin lysates of young and old mice. We observed a significant increase in the expression of p16 in aged murine skin (Figure 1g), alongside elevation in several SASP factors (Figure 1i), while the expression of p21 remained unchanged (Figure 1h).

### 2.2 Transplanting senescent cells intradermally induces paracrine senescence and age‐related dysfunction

Following our observation that senescence‐associated markers increased in mouse skin during aging, we sought to test whether senescent cells in the skin can have systemic effects in other organs. To achieve this, we conducted experiments involving the transplantation of senescent or proliferating (non‐senescent) adult fibroblasts isolated from the ears of syngeneic luciferase‐expressing transgenic (LUC+) mice into the dermis of young (3‐month‐old) wild‐type mice (Figure S1a). Senescence was induced in dermal fibroblasts by exposure to 10Gy x‐ray irradiation, resulting in increased expression of multiple senescence‐associated markers, including SA‐β‐Gal, γH2A.X, expression of p21, p16 and various SASP factors, and a decrease in Ki67 (Figure S1b–h). Subsequently, both proliferating and senescent fibroblasts were intradermally transplanted (Figure S1i), and their presence and kinetics was monitored through in‐vivo bioluminescence imaging. We observed that both t‐Prolif and t‐Sen cells gradually decreased over the course of 22 days, with similar clearance rates, eventually becoming virtually undetectable (Figure S1j,k). Two months post‐transplantation, we assessed potential migration of these transplanted cells in the skin to other tissues; however, no luciferase signal was detected in any other organs (Figure S1l).

We also noted that intradermal injection of senescent cells did not affect body weight (Figure S2a). However, we observed an increase in the weight of the heart and liver upon normalization to body weight (Figure S2b–d).

**FIGURE 2:**
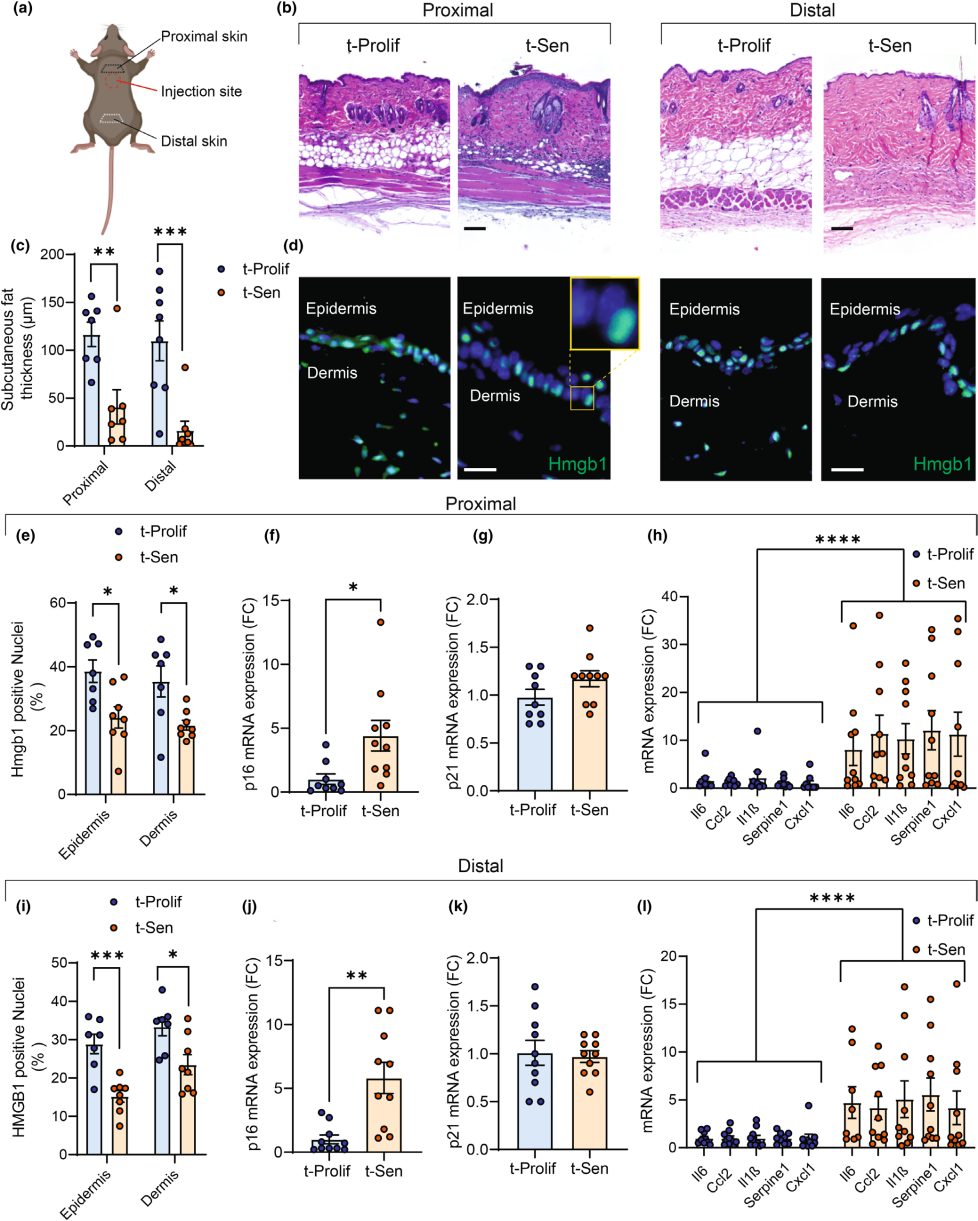
Transplanting senescent cells intradermally induces paracrine senescence in skin. (a) 5 months after transplantation skin was collected in regions proximal or distal from injection site. (b) Representative images of H&E micrographs from skin located proximally or distally from injection site (scale bar 50 μm). (c) Quantification of subcutaneous far thickness in distal or proximal skin; (d) representative images of HMGB1 immunostaining in proximal (left) and distal (right) transplanted skin (scale bar 20 μm). (e) Quantification of HMGB1 positive nuclei in the epidermis and dermis of proximal skin. (f–h) q‐PCR analysis of p16, p21 and SASP factors in proximal transplanted skin. (i) Quantification of HMGB1 positive nuclei in the epidermis and dermis of distal skin. (j–l) q‐PCR analysis of p16, p21 and SASP factors in distal transplanted skin. Data are expressed as the mean ± SEM. *N* = 7–10 per group. *p < 0.05, **p < 0.01, ***p < 0.001, ****p < 0.0001 significantly different compared to t‐Prolif mice, as determined by Student's *t*‐test.

Five months after transplantation, we examined the skin tissue and subcutaneous fat located proximal or distal to the cell transplantation site (Figure 2a). Skin thinning and loss of subcutaneous fat are common features of aging (Farage et al., 2013). We observed unchanged epidermal and dermal thickness (Figure S3a,b), but a notable reduction in subcutaneous fat thickness in skin both proximal and distal to the cell transplantation site (Figure 2b,c). We also observed an increase in CD45 and CD68 immune cells in skin proximal to injection site (Figure S3c). We then investigated whether intradermal transplantation of senescent cells had an impact on senescence markers in the skin in areas proximal or distal to the injection site. HMGB1 is known to migrate from the nucleus to the extracellular milieu in senescent cells (Davalos et al., 2013). We found that transplantation of senescent cells resulted in a decrease in the frequency of HMGB1 positive nuclei in the epidermis and dermis regardless of proximity to the injection site (Figure 2d,e,i). Additionally, we found that transplantation of senescent cells led to an increase in the expression of senescence‐associated marker p16 (but not p21) and several SASP factors proximally or distally from the injection site (Figure 2f–h,j–l). These results provide support for the hypothesis that senescent cells have the capacity to spread senescence or age‐related dysfunction across tissues and are consistent with studies in which transplanting other types senescent cells, tissue explants, or tissues or organs from older donors led to development of senescent cells in organs distant from the transplantation site (Iske et al., 2020; Xu et al., 2018).

Given that other studies have suggested that paracrine senescence is mediated via the SASP (Acosta et al., 2013), we evaluated secreted factors in plasma 5 months post‐transplantation using a multiplex cytokine array consisting of 32 different factors. While no significant differences were observed in most factors analyzed, we did observe a tendency for an increase in G‐CSF and a significant increase in IL‐6 in plasma from mice following t‐Sen transplantation (Figure S4a,b).

**FIGURE 3:**
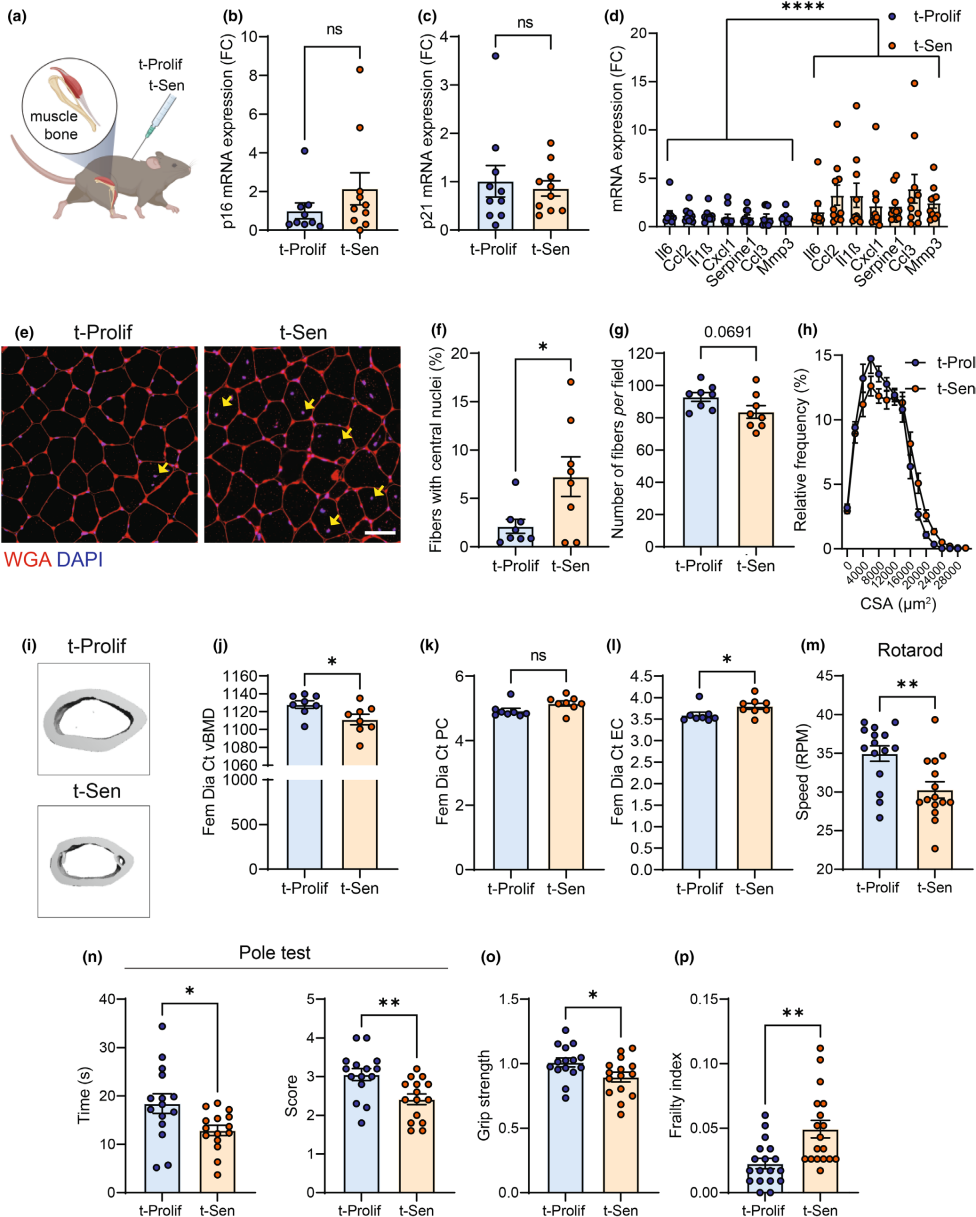
Transplanting senescent cells intradermally induces age‐associated parameters in skeletal muscle and bone. (a) 5 months following intradermal transplantation, muscle (quadriceps) and bone were collected. mRNA levels of senescence‐associated markers (b) p16; (c) p21 and (d) several SASP factors were analyzed in skeletal muscle by q‐PCR. (e) representative images of WGA staining in skeletal muscle of mice transplanted with either proliferating or senescent cells (scale bar 60 μm). Yellow arrows denote centrally nucleated fibers. Quantification of (f) of % of centrally nucleated fibers; (g) number of fibers per field and (h) fiber cross‐sectional area (CSA) distribution. (i) Representative reconstructed μCT images of bone microarchitecture of femur cortical bone (diaphysis) 5 months after transplantation of t‐Prolif and *t*‐Sen. Quantification of (j) femoral diaphysis cortical volumetric bone mineral density (Ct.vBMD) measured in mg.cm‐3 (k and l), Quantification of femoral diaphysis periosteal circumference and endocortical circumference. (m) 4 months after transplantation, mice were assessed for motor coordination using the rotarod test (n) Pole test, (o) forelimb grip strength and (p) multiple parameters of clinical frailty. Data are represented as mean ± SEM (*n* = 8–15 animals per group). Statistical differences *p < 0.05, **p < 0.01, and ****p < 0.0001 were by Students *t*‐test and two‐way ANOVA compared with t‐Prolif controls.

We then analyzed skeletal muscle and bone structure in these animals (Figure 3a). We observed a trend towards increased p16 expression, but not p21 (Figure 3b,c) and increased expression of SASP factors in skeletal muscle of mice receiving senescent cell transplants (Figure 3d). Additionally, mice transplanted with senescent cells exhibited an elevated occurrence of centrally nucleated fibers in skeletal muscle (Figure 3e,f), together with a tendency for decreased numbers of muscle fibers *per* field (Figure 3g), while the cross‐sectional area of these fibers remained unchanged (Figure 3h). Finally, micro‐computed tomography (μCT) analysis of bone revealed that dermal transplantation of senescent cells led to a decreased femoral diaphysis cortical mineral density together with an increased diaphysis endocortical circumference which is an indication of bone loss (Figure 3i–l).

Four months after intradermal transplantation, we performed several tests to assess physical function and frailty in these mice. We found that mice transplanted with senescent cells, showed a significant impairment in motor coordination measured using the RotaRod test (Figure 3m), were less capable of maintaining balance on a raised rod (Figure 3n) and showed reduced forelimb grip strength (Figure 3o). We then assessed multiple parameters of clinical frailty (Whitehead et al., 2014), known to increase with age and found that frailty index was significantly higher in mice that had been intradermally transplanted with senescent cells (Figure 3p). Together, these findings suggest that intradermal transplantation of senescent cells impaired motor coordination, musculoskeletal function, and frailty in mice.

### 2.3 Transplanting senescent cells intradermally induces senescence in the hippocampus

**FIGURE 4:**
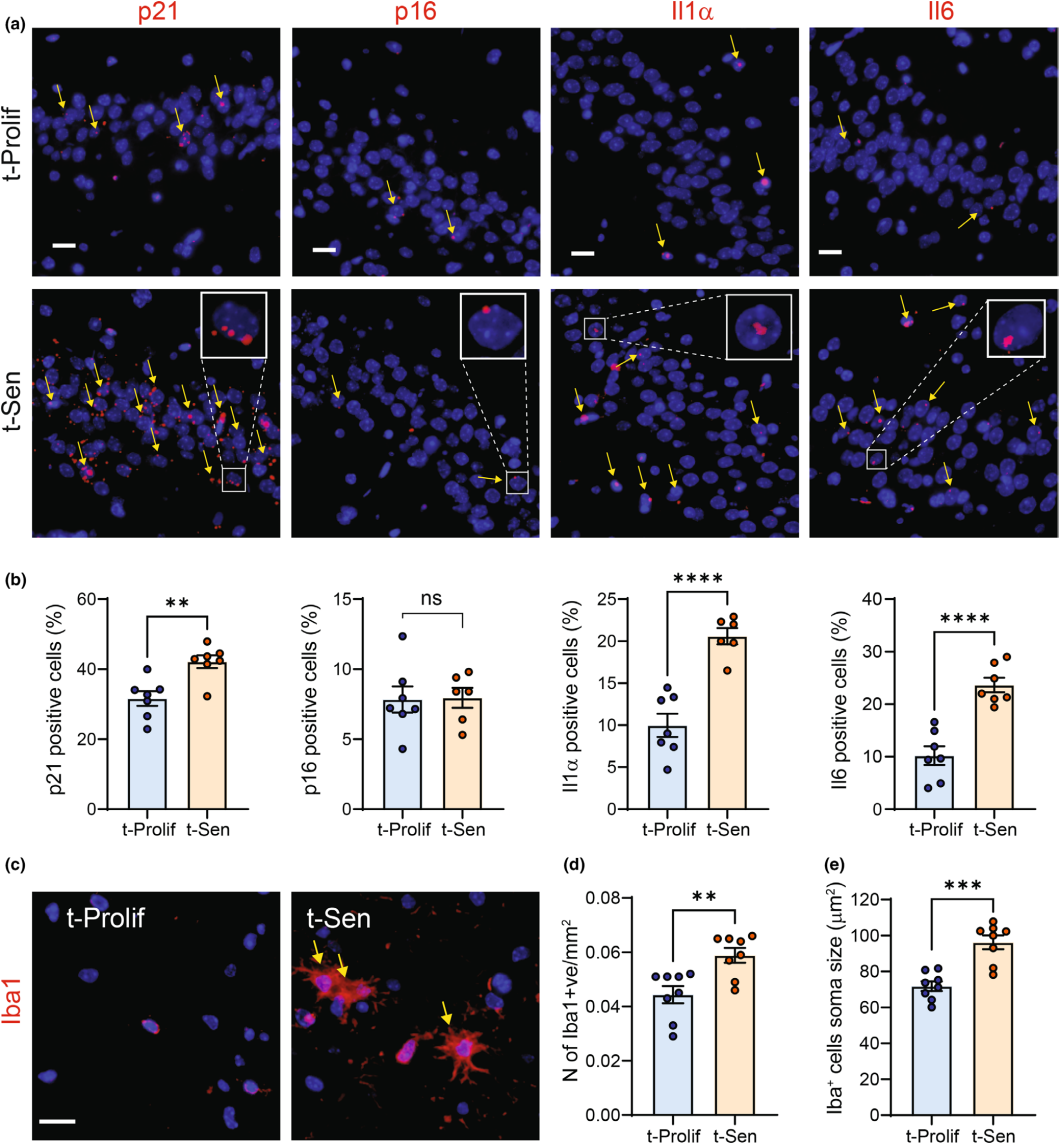
Transplanting senescent cells intradermally induces paracrine senescence in host hippocampus. (a) Representative images and quantification of RNA‐ISH detection of p21, p16, Il1α and Il6 in the CA3 region of the hippocampus of transplanted mice (Scale Bar 20 μm). Yellow arrows indicate positive cells. (b) Quantification of the percentages of positive cells for p21, p16, Il1α and Il6 in the respective hippocampal region. (c) Representative images of Iba1 immunostaining in the hippocampus of transplanted mice. Yellow arrows indicate activated Iba1+ microglia (Scale Bar 20 μm). Quantification of the number of Iba1 positive cells d) and microglia's soma size (an indication of activation) (e). Data are represented as mean ± SEM (*n* = 8 animals per group). Statistical differences **p < 0.01, ***p<0.001 and ****p < 0.0001 were by Students *t*‐test compared with t‐Prolif controls.

Given the known accumulation of senescent cells in the hippocampus of aged mice and the improvement in cognitive function upon clearance of these cells (Ogrodnik et al., 2021), we investigated senescence‐associated markers in this brain region, crucial for memory function and cognition. Employing RNA‐ISH, we observed a significant increase in p21^Cip1a^‐positive cells (but not p16^Ink4a^) in the CA3 region of the hippocampus, alongside an increased frequency of cells expressing SASP factors Il‐1α and Il‐6 in mice transplanted with senescent cells in the skin (Figure 4a,b).

Furthermore, immunostaining for the microglia marker Iba1 revealed a significant higher number and soma size of microglia in mice transplanted with senescent cells, indicative of microglial activation (Figure 4c–e), a phenotype commonly observed during aging (Fielder et al., 2020; Ogrodnik et al., 2021). These data imply that skin senescence may lead to secondary senescence in the brain through yet unidentified mechanisms.

### 2.4 Transplanting senescent cells intradermally induces cognitive decline in young mice

**FIGURE 5:**
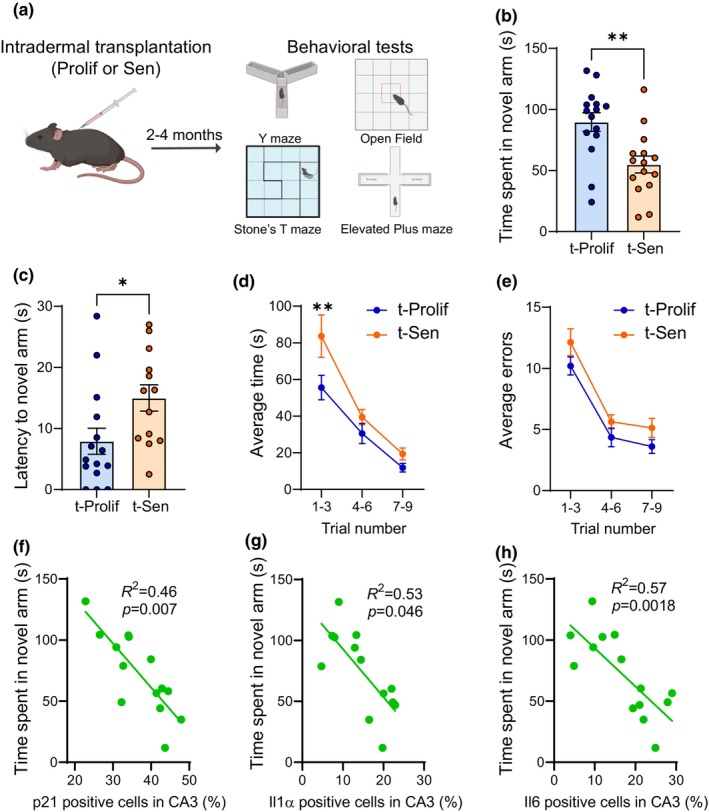
Transplanting senescent cells intradermally induces cognitive decline in young mice. (a) 2–4 months following intradermal transplantation of proliferative or senescent fibroblasts, mice were evaluated a series of behavioral assessments including the Y‐maze and Stones maze tests, which assess spatial memory, as well as the Elevated Plus Maze (EPM) and Open Field tests, which assess anxiety‐like behavior. Using Y‐maze, transplanted mice were evaluated for (b) Time spent in novel arm and (c) latency to novel arm. Using Stone's maze transplanted mice were assessed for (d) average time to finish the test and (e) average number of errors. Linear regression analysis of time spent exploring Y maze's open arm and (f) p21, (g) IL1α and (h) Il6 positive cells in the hippocampus CA3 region. Data are mean ± S.E.M of *n* = 15 animals per group. *p < 0.05, **p < 0.01.

Multiple studies have consistently shown that aging is accompanied by a decline in cognitive function (Bettio et al., 2017; Fielder et al., 2020; Ogrodnik et al., 2021). To explore whether the intradermal transplantation of senescent cells induces cognitive decline in young mice, we conducted a series of behavioral tests, including Y‐maze and Stones T‐maze, which evaluate spatial memory. Moreover, we also assessed the anxiety‐like behavior using the Elevated Plus Maze (EPM) and Open Field tests (Figure 5a). Our findings revealed that intradermal transplantation of senescent cells led to a substantial reduction in the time spent in the novel arm and an increase in the latency before entering the novel arm as determined by the Y‐maze test (Figure 5b,c). Additionally, using the Stone T‐maze, we observed that mice transplanted with senescent cells required more trials to complete the maze and tended to commit more errors before successfully navigating to the exit (Figure 5d,e). These results indicate cognitive decline which parallels previous observations in aged mice (Ogrodnik et al., 2021). Interestingly, our analysis of anxiety‐like behavior using the EPM and Open Field tests did not reveal any significant differences between mice receiving senescent cell transplants and control groups (Figure S5a,b), indicating that intradermal senescent cell transplantation does not induce anxiety‐related behaviors.

To assess whether senescence‐associated markers in muscle and brain correlate with functional outcomes, we conducted a series of correlation analyses. Our analyses did not reveal any significant correlations between senescence markers in skeletal muscle and measures of muscle function, such as the rotarod test, pole test, or grip strength (Figure S6). Consistent with links between senescence and cognitive impairment, we found inverse correlations between time spent exploring Y maze's open arm and the frequency of p21, Il1α and Il6 positive cells in the hippocampus (Figure 5f–h).

## 3 DISCUSSION

Skin aging can result from two main factors: intrinsic aging, and extrinsic aging caused by environmental factors which can ultimately compromise the structural integrity and physiological function of the skin (Wyles et al., 2024). Cellular senescence has been found to have beneficial effects in skin wound healing (Demaria et al., 2014) and in preventing cancer development (Michaloglou et al., 2005). While short‐term induction of cellular senescence can be advantageous in these scenarios, the accumulation of senescent cells can be harmful due to the chronic secretion of SASP factors, which can exacerbate tissue dysfunction. Studies have revealed that senescent cells accumulate during aging in murine, primate (Herbig et al., 2006) and human skin (Victorelli et al., 2019). Chronic exposure to UV light has been shown to induce senescence in different skin cell‐types (Debacq‐Chainiaux et al., 2012; Victorelli et al., 2019). Human 3D skin equivalents containing senescent cells experience paracrine senescence and reduced proliferation, leading to epidermal thinning, a process alleviated by senolytic drugs (Victorelli et al., 2019). Similarly, senolytics rescued the proliferation of hair follicle stem cells in a transgenic mouse model where senescence was specifically induced in the basal layer of the epidermis (Yosef et al., 2016). These findings show that the accumulation of senescent cells contributes to skin aging.

Studies have uncovered broader implications of skin aging, indicating a significant link between reduced skin wrinkling in sun‐protected regions and familial longevity (Gunn et al., 2013), along with a positive correlation between perceived age and survival (Gunn et al., 2016). The p16^INK4a^ positivity in the skin was associated with the cardiovascular disease‐risk (Gunn et al., 2013; Waaijer et al., 2012) and immunosenescence (Waaijer et al., 2019). Yet, a causal link between skin senescence and other age‐related dysfunction and diseases, particularly neurocognitive diseases, remains unclear, as does the direction of this relationship.

Additional evidence points to a potential association between skin aging and central nervous system function. UV‐induced skin aging in mice resulted in neurological and hippocampal impairment, marked by diminished levels of neurotrophic factors, synaptic proteins, and neurogenesis within the hippocampus (Han et al., 2017). Moreover, chronic UV exposure led to cognitive decline, decreased expression of neuroimmune system elements, and microglial activation (Yoon et al., 2022), indicating a potential interplay between skin aging and CNS integrity. However, the specific involvement of UV‐induced senescent cells in mediating these effects remains unclear.

Both the brain and skin develop from the embryonic ectodermal layer. (Paré & Gros‐Louis, 2017) Perhaps related to this, it appears a range of brain and skin diseases may be linked, such as in the case of chronic UV exposure above as well as the skin changes associated with amyotrophic lateral sclerosis (Paré & Gros‐Louis, 2017). Another example is the link between psoriasis and Alzheimer's disease, with some evidence suggesting an increased risk for patients with psoriasis to develop Alzheimer's disease as well as for patients with Alzheimer's disease to develop psoriasis (Kim et al., 2024). Of note, both psoriasis (Mercurio et al., 2020) and Alzheimer's disease (Gonzales et al., 2022) are associated with increased senescent cell burden at sites of pathology. Yet another example is the link among cellular senescence, Parkinson's disease, and the skin cancer, melanoma (Bose et al., 2018). Although the risk of most cancers is decreased in patients with Parkinson's disease, the risk for melanoma is strongly increased: there is a 2‐to‐20‐fold greater risk of melanoma in patients with Parkinson's disease. Additionally, there is a 50% to over fourfold higher risk for Parkinson's disease in patients with melanoma. Senescent cell abundance is increased both in the substantia nigra in Parkinson's disease (Bose et al., 2018) and around sites of melanoma (Liu et al., 2023).

Perhaps explaining links between age‐ and damage‐related skin disorders and brain diseases, we found that transplanting senescent fibroblasts into the skin leads to decreased musculoskeletal function, frailty, and neurocognitive dysfunction, all of which are common aging phenotypes. These findings support the hypothesis that cellular senescence may be a root cause mechanism linking skin diseases and neurocognitive dysfunction. Notably, we observed a decrease in performance in Y‐maze and Stone's maze tasks following the transplantation of senescent cells, accompanied by microglia activation and heightened levels of senescence‐associated markers in the hippocampus, mirroring observations made during the aging process (Ogrodnik et al., 2021). This suggests that factors secreted by senescent cells located in the skin may have broad‐reaching effects. However, further research is needed to pinpoint which factors released by senescent cells in the skin drive the systemic effects observed in host tissues, potentially opening avenues for therapeutic intervention.

During aging, different organs exhibit senescence markers in a heterogeneous and tissue‐specific manner (Saul et al., n.d.). Our study shows that aged skin displays increased p16 expression, while previous research has found that neurons in the CA3 region of the hippocampus predominantly exhibit elevated p21 levels (Ogrodnik et al., 2021). These findings highlight the heterogeneity and distinct roles of p21 and p16 in senescence across cell types. Consistently, our results indicate that paracrine senescence varies by tissue, with the skin linked to increased p16 but not p21, and the hippocampus showing higher p21 but not p16.

A limitation of our study is that the amount and composition of transplanted senescent cells does not accurately reflect senescent cell accumulation during physiological aging. Future research should include other models of senescence induction in the skin, including exposure to physiological levels of UV irradiation. Furthermore, to determine whether paracrine senescence is the causal factor driving the observed aging phenotypes, experiments involving the clearance of senescent cells using senolytic drugs or genetic models that enable the removal of p16 or p21 positive cells should be conducted.

In addition, further research is needed to pinpoint which factors released by senescent cells in the skin drive the systemic effects observed in host tissues. Such mechanistic studies could open new avenues for therapeutic intervention.

Our study offers proof‐of‐concept evidence that senescent cells in the skin may exert widespread age‐related effects on other organs, especially the brain.

## 4 MATERIALS AND METHODS

### 4.1 Cell culture

Ear clippings from Luciferase transgenic C57BL/6 mice were transported in DMEM containing serum on ice. Punches were washed three times with serum‐free media, finely cut and incubated for 2–3 h at 37°C in 2 mg.mL^−1^ collagenase A in DMEM. A single‐cell suspension was obtained by repeated pipetting and passing through a 24G fine needle. Cells were centrifuged for 10 min at 96 rcf and cultured in Advanced DMEM/F‐12 (DMEM, Invitrogen) plus 10% fetal calf serum (Sigma) in 3% O_2_ and 5% CO_2_. Cells were x‐ray‐irradiated with 10 Gy. Immediately following irradiation, media were replaced, and cells were cultured for an additional 10 days and then analyzed for senescence‐associated markers.

### 4.2 Animals

All animal experiments were conducted in accordance with protocols approved by the Institutional Animal Care and Use Committee (IACUC) at Mayo Clinic. Wild‐type male 3‐month‐old C57BL/6 mice were purchased from Jackson laboratories and housed in a pathogen‐free facility with controlled conditions, including a temperature range of 23–24°C and a 12‐h light–dark cycle. These mice were provided with ad libitum access to a standard mouse diet (Lab Diet 5053, St. Louis, MO) and water, with quarterly pathogen testing consistently yielding negative results. Mice were maintained on a normal chow diet and housed in static autoclaved HEPA‐ventilated microisolator cages (27 × 16.5 × 15.5 cm) containing autoclaved Enrich‐o'Cobs bedding. Bedding and cages were changed biweekly within class II biosafety cabinets.

Additionally, luciferase transgenic C57BL/6 mice (LUC+), expressing firefly luciferase under the constitutively active CAG promoter in most tissues, were obtained from The Jackson Laboratory (Bar Harbor, ME; stock no. 025854).

### 4.3 Intradermal transplantation of senescent or proliferating fibroblasts

Fibroblasts previously isolated from the ear skin of LUC+ animals were cultured followed by exposure to x‐ray irradiation at a dose of 10 Gy to induce senescence. After irradiation, cells were collected for transplantation on Day 10. Control cells were cultured without irradiation. Senescent or control cells were harvested by trypsinization, washed once with PBS, and resuspended in PBS for transplantation. For cells transplantation, 3‐month‐old C57BL6 mice were anesthetized using isoflurane, and 1 × 10^6^ senescent or control cells in 100 μL were injected intradermally at two points in the anterior part of the animal's body, using a 31‐G insulin needle, into previously shaved skin.

### 4.4 Bioluminescence imaging

Mice received an intraperitoneal injection of 3 mg D‐luciferin (Gold Biotechnology, St. Louis, MO) dissolved in 200 μL PBS. Subsequently, mice were anesthetized with isoflurane, and bioluminescence images were captured using a Xenogen Ivis 200 System (Caliper Life Sciences, Hopkinton, MA) following the manufacturer's guidelines.

### 4.5 Y maze

Spatial and working memory assessments in mice were conducted using a Y maze setup (Fielder et al., 2020). The Y maze comprises three arms with elevated walls. The animals were habituated to the room at least 1 h prior to testing. In the initial training trial, one arm, designated as the novel arm, was blocked, allowing the mouse to explore the remaining arms freely for a duration of 5 min. Subsequently, in the test trial, the mouse had unrestricted access to all three arms while tracked by camera with tracking software (Ethovision). The evaluation involved determining spontaneous alternation, quantified by the number of entries made into the novel arm, and time spent exploring the novel arm.

### 4.6 Stone T‐maze

A water‐motivated adaptation of the Stone T‐maze (custom‐made by Mayo Clinic workshop) was employed to evaluate cognitive function, a method shown to reveal age‐related learning and memory deficits. This maze, featuring an acrylic‐roofed structure, is set within a shallow steel pan filled with water, prompting mice to navigate towards a dry escape zone while maintaining their heads above water. Initially, mice underwent a day of straight run training to grasp the concept of reaching the goal box for escape. The Stone T‐maze testing commenced the following day, with each mouse undergoing 9 acquisition trials. Intertrial intervals of 15 min were provided in a holding cage equipped with a heat lamp. Key metrics of learning and memory include the latency to reach the goal box and the occurrence of errors, defined as complete entry of the mouse's head into an incorrect path. Failure to reach the goal box within 6 min resulted in a trial being marked as unsuccessful, with three consecutive failures leading to exclusion. Following testing, mice were dried under a heat lamp.

### 4.7 Elevated plus maze

Anxiety and locomotor activity were assessed by the Elevated Plus Maze test. The Elevated Plus Maze apparatus comprises two open arms (36 × 6 cm) and two enclosed arms (36 × 6 cm) positioned perpendicular to each other, all attached to a central platform elevated 75 cm above ground level. Prior to testing, the animals underwent habituation to the testing room for at least 1 h. Individually, mice were positioned at the center of the maze facing an open arm and allowed to explore freely for 5 min, with their movements tracked by camera using tracking software (Ethovision). Anxiety levels were assessed by analyzing the frequency and duration of time spent in the open arms.

### 4.8 Open field

The locomotor activity and anxiety‐like behavior of mice were evaluated in activity chambers (Med Associates, St Albans, VT, USA: dimensions 27 cm × 27 cm × 20 cm) equipped with continuous fans, infrared lasers, and sensors for accurate monitoring. Beam breaks were analyzed in 2‐min intervals over a 30‐min period, automatically converting data to mouse location and distance traveled (in cm), recorded using Med‐PC software Version 4.0 on a computer. Prior to testing, mice were allowed to acclimate to the testing environment for 1 h. Subsequently, they underwent a 5‐min habituation period in the Open Field chamber (without recording), followed by another 5 min in their cage. Afterwards, mice were reintroduced to the chambers, and their movements were recorded for 30 min. Anxiety levels were assessed by measuring the distance traveled by mice within the central 25% of the chamber (zone 1) relative to the total distance traveled, as well as by analyzing the frequency of entries into zone 1.

### 4.9 RotaRod

Assessment of maximal walking speed and latency was performed using an accelerating RotaRod system (Ugo Basile, Rota Rod 47650). Mice were trained on the RotaRod for 3 consecutive days prior to test day. Training consisted of mice remaining on RotaRod at speeds of 4, 6 and 8 rpm for 300 s on Days 1, 2, and 3, respectively. On the test day, mice were placed on the rotating cylinder, which increased in speed from 4 to 40 rpm over a 300 s interval. The speed and time at which a mouse fell off the cylinder were recorded. Results were the average of three trials.

### 4.10 Pole test

Assessment of balance and motor coordination were done using Pole test (Correia‐Melo et al., 2019). Animals were placed on a horizontal bar, which was 60 cm length and situated 50 cm off the ground. The time animals were able to spend on top of the bar was recorded. A trial was deemed successful if an animal could remain on top of the bar for 60 s without falling. Each mouse was given five trials with a 30 s rest between trials.

### 4.11 Forelimb grip strength analysis

Forelimb grip strength was assessed utilizing a Grip Strength Meter. In this procedure, mice briefly grasped a bar connected to a computer‐integrated force transducer using their front limbs, after which they were gently pulled away from the bar. Three trials were conducted for each mouse, and the average peak force will be calculated.

### 4.12 Frailty measurements

Frailty was assessed using a 30‐parameter index as previously described (Whitehead et al., 2014). For each parameter, mice were given a score of 0, 0.5 or 1 corresponding to absence, mild or severe phenotype, respectively. Body weight was recorded, and surface body temperature was measured using an infrared temperature probe. For dystonia assessment, a score of 1–4 was given, where a score of 1 was equivalent to clasping with one limb whereas a score of 4 was given if the animal showed clasping with all four limbs.

### 4.13 Skeletal imaging

All bone imaging and analysis was done in a blinded fashion. Quantitative ex vivo analyses of bone microarchitecture of the femur (proximal metaphysis/mid‐shaft diaphysis) were performed as before (Victorelli et al., 2023) using a μCT system. This analysis was conducted using the manufacturer's software provided by Scanco Medical AG in Bassersdorf, Switzerland, with Finite Element‐Software Version 1.13. Scan settings were as follows: 55kVp, 145 mA, high resolution, 21.5 diameter, 10.5 μm voxel size, 300 ms integration time. At the proximal metaphysis and mid‐diaphysis (50 slices) of the femur, cortical thickness (Ct.Th; mm), endocortical circumference (E.C; mm) and periosteal circumference (P.C; mm) were assessed.

### 4.14 Q‐PCR

Total RNA from homogenized tissue biopsies or cells was isolated using TRI Reagent (Sigma‐Aldrich) or the RNAeasy Mini Kit (Qiagen, 74106) according to the manufacturer's instructions Complementary DNAs were synthesized using the High‐Capacity cDNA Reverse Transcription Kit (ThermoFisher, 4,368,814) following manufacturer's instructions. Quantitative real‐time PCR was carried out using either Power SYBR® Green PCR Master Mix (Invitrogen, 4,367,659) in a C100TM Thermal Cycler (BioRad), ToughMix Perfecta (PerfeCTa qPCR ToughMix, QuantaBio, 95,112–250) using CFX96TM Real‐Time System (Bio‐Rad), Brilliant III Ultra‐Fast SYBR Green qPCR Master Mix (Agilent Technologies).

Real‐Time PCR System (Bio‐Rad), and Bio‐Rad CFX Manager software. Predesigned primers and probes from IDT PrimeTime. Mouse: p16: Mm.PT.58.42804808; p21: Mm.PT.58.5884610; IL6: Mm.PT.58.13354106; CCL2: Mm.PT.58.42151692;CCL3: Mm.PT.58.29283216;CCL5: Mm.PT.58.434548565; IL1α: Mm.PT.58.32778767; IL1β:Mm.PT.58.41616450; Serpine1: Mm.PT.58.6413525; TNF: Mm.PT.58.12575861; Cxcl1: Mm.PT.58.42076891; MMp13: Mm.PT.58.42286812; HPRT: Mm.PT.39a.22214828.mRNA levels were calculated using the 2‐ΔΔCT method and normalized to a housekeeping gene.

### 4.15 SA‐beta‐galactosidase assay

Mouse Adult Fibroblasts (MAFs) cultured on coverslips were fixed in 4% paraformaldehyde (PFA) in PBS for 10 min. Subsequently, they were stained with Sen‐β‐Gal staining solution using the Senescence β‐Galactosidase Staining Kit (Cell Signaling Technology #9860) according to manufacturer's instructions. After incubation, the cells were washed three times with PBS and then mounted onto glass microscope slides using ProLong Gold Antifade Mountant with DAPI (Invitrogen).

### 4.16 Immunocytochemistry and immunohistochemistry

Cells grown on coverslips were fixed in 4% paraformaldehyde in PBS for 10 min. Cells were then permeabilized in PBG‐Triton (PBS, 1% BSA, 0.5% Triton X‐100) for 45 min and incubated with primary antibody overnight at 4°C with anti‐ki67 (ab15580) rabbit polyclonal (1:200, Abcam) and anti‐Phospho‐Histone H2A.X (Ser139), Clone JBW301 (05–636) mouse monoclonal (1:200, Millipore). Following PBS washes, cells were incubated with secondary antibody for 45 min and mounted onto glass microscope slides with ProLong Gold Antifade Mountant with DAPI (Invitrogen).

Formalin‐fixed paraffin‐embedded (FFPE) tissue sections (5 μm) were deparaffinized in Histoclear (5 times for 5 min each) and hydrated using 100% ethanol (twice for 5 min), 90% ethanol (twice for 5 min), 70% ethanol (5 min) and distilled water (twice for 5 min each). Antigen retrieval was done by heating to boiling in citrate buffer pH 6.0 (Agilent‐Dako, S236984) for 10. Slides were allowed to cool down for 30 min and were then rinsed in PBS twice for 5 min. Tissue sections were blocked with 1%BSA/1:60NGS (Agilent‐Dako, X090710‐8) for 30 min at room temperature followed by primary antibody incubation overnight at 4°C. Sections were washed in PBS and incubated with secondary antibody for 1 h at room temperature. After 3 PBS washes sections were mounted with ProLong Gold Antifade Mountant with DAPI (Invitrogen). The primary antibodies used were as follows: Anti‐phospho‐Histone H2A.X (Ser139) Antibody, (9718S) rabbit monoclonal (1:200, Cell Signalling); Anti‐PCNA antibody [PC10] (ab29) mouse (1:500, Abcam); Anti‐Iba1 antibody (NB100‐1028) goat (1:200, Novus Biologicals); Anti Lamin B1 (ab16048) rabbit polyclonal (1:200, Abcam).

### 4.17 RNA‐ISH

RNA‐ISH was performed according to RNAscope protocol from Advanced Cell Diagnostics (ACD). Paraffin sections were deparaffinized with Histoclear (twice 5 min) and rehydrated in 100% ethanol (EtOH) (twice 1 min). Sections were allowed to air dry and then incubated with H_2_O_2_ for 10 min at room temperature followed by another 2 washes in H_2_O. Sections were placed in hot 1X antigen retrieval solution and boiled for 15 min. After washes in H_2_O and 100% EtOH, sections were air dried. Sections were treated with protease plus for 30 min at 40°C, washed with H_2_O, and incubated with target probes: p21 (#408551), p16 (#411011), IL1α (#440391) or IL‐6 (#315891) for 2 h at 40°C. Next, slides were washed with H_2_O followed by incubation with AMP1 (30 min at 40°C) and next washed with wash buffer (WB) and AMP2 (15 min at 40°C), WB and AMP3 (30 min at 40°C), WB and AMP4 (15 min at 40°C), WB and AMP5 (30 min at RT) and WB, and, finally, AMP6 (15 min at RT). Lastly, an RNAscope 2.5 HD Reagent kit‐RED was used for chromogenic labelling. Sections were washed in H_2_O five times and were then mounted using ProLong Gold mounting media containing DAPI.

### 4.18 Immuno‐FISH

Sections of formalin‐fixed paraffin‐embedded tissue were deparaffinized in 100% Histoclear, gradually hydrated through a series of ethanol concentrations (100%, 90%, and 70% ethanol, twice for 5 min each), and rinsed twice for 5 min in distilled water. Antigen retrieval was carried out by immersing the sections in 0.01 M citrate buffer (pH 6.0) and heating to boiling for 10 min. Following cooling to room temperature, the sections were washed in distilled water for 5 min. Blocking was then performed using normal goat serum (1:60) in BSA/PBS for 30 min. Samples were further blocked with Avidin/Biotin (Vector Lab, Burlingame, CA) for 15 min at room temperature each, followed by overnight incubation with rabbit monoclonal anti‐γH2AX antibody (1:200, 9718; Cell Signaling) at 4°C. After three washes in PBS, the tissues were incubated with a goat anti‐rabbit biotinylated secondary antibody (1:200, PK‐6101; Vector Labs) for 30 min at room temperature. Subsequently, the sections were washed three times in PBS and incubated with fluorescein Streptavidin Cy5 (1:500, A‐2011; Vector Labs) for 30 min at room temperature. The tissues were further washed three times in PBS and incubated in 4% paraformaldehyde in PBS for 20 min for cross‐linking. After three PBS washes, the sections were dehydrated in graded cold ethanol solutions (70%, 90%, 100%) for 3 min each and allowed to air dry. Then, 10 μL of PNA hybridization mix (70% deionized formamide, 20 mM MgCl2, 1 M Tris pH 7.2, 5% blocking reagent containing 2.5 μg/mL Cy‐3‐labelled telomere‐specific (CCCTAA) peptide nucleic acid probe (PANAGENE)) was added to the sections, and denaturation occurred for 10 min at 80°C. Subsequently, the sections were incubated in the PNA hybridization mix for 2 h at room temperature in the dark to enable hybridization. Tissues were washed in 70% formamide in 2× SSC for 10 min, followed by one wash in 2× SSC for 10 min and a PBS wash for 10 min. Finally, tissues were mounted using ProLong Gold Antifade Mountant with DAPI (Invitrogen) and imaged using in‐depth z stacking (a minimum of 40 optical slices with a × 63 objective).

### 4.19 Cytokine array

Cytokines and chemokines in mouse plasma were detected using Eve Technologies' Mouse Cytokine/Chemokine 32‐Plex Discovery Assay® Array (MD32).

### 4.20 Microscopic imaging

Microscope imaging was performed using an epifluorescence brightfield Leica DMi8 inverted microscope. ImageJ software was used for the quantification of histomorphometric parameter and positive cells for each specific cell marker.

### 4.21 Statistical analysis

All results are presented as mean ± SEM. Graphs were made, and statistical analyses were done using in Graph Pad Prism 8 software. Statistical differences were calculated by unpaired *t*‐tests and One‐way ANOVA; values of *p* < 0.05 were considered statistically significant (*p < 0.05, **p < 0.01, ***p < 0.001, and ****p < 0.0001 as indicated in the respective figure legends).

## AUTHOR CONTRIBUTIONS

ACF performed most of the experiments; HM, SV, ABL, NP, SPW, SW, DGC performed and evaluated individual experiments; JLK, SGT, DJ, and CC supervised individual experiments; CC and JFP designed and supervised the study; JFP and ACF wrote the manuscript with the contributions from all the authors.

## CONFLICT OF INTEREST STATEMENT

JLK and TT, and SGT have a financial interest related to this research. Patents on senolytic drugs are held by Mayo Clinic and Brigham and Women's Hospital. This research has been reviewed by the Mayo Clinic Conflict of Interest Review Board and was conducted in compliance with Mayo Clinic Conflict of Interest policies. The remaining authors declare no competing interests.

## Supporting information


Figure S1.

## Data Availability

The data that support the findings of this study are available from the corresponding author upon reasonable request.
