# Supplementary material for: Senescent cell transplantation into the skin induces age‐related peripheral dysfunction and cognitive decline
Source: Aging Cell. 2024 Oct 7;24(1):e14340. doi: 10.1111/acel.14340 (PMC11709089; doi:10.1111/acel.14340)
Supplement: Supplementary file 1 — Figure S1. [file ACEL-24-e14340-s001.pdf]

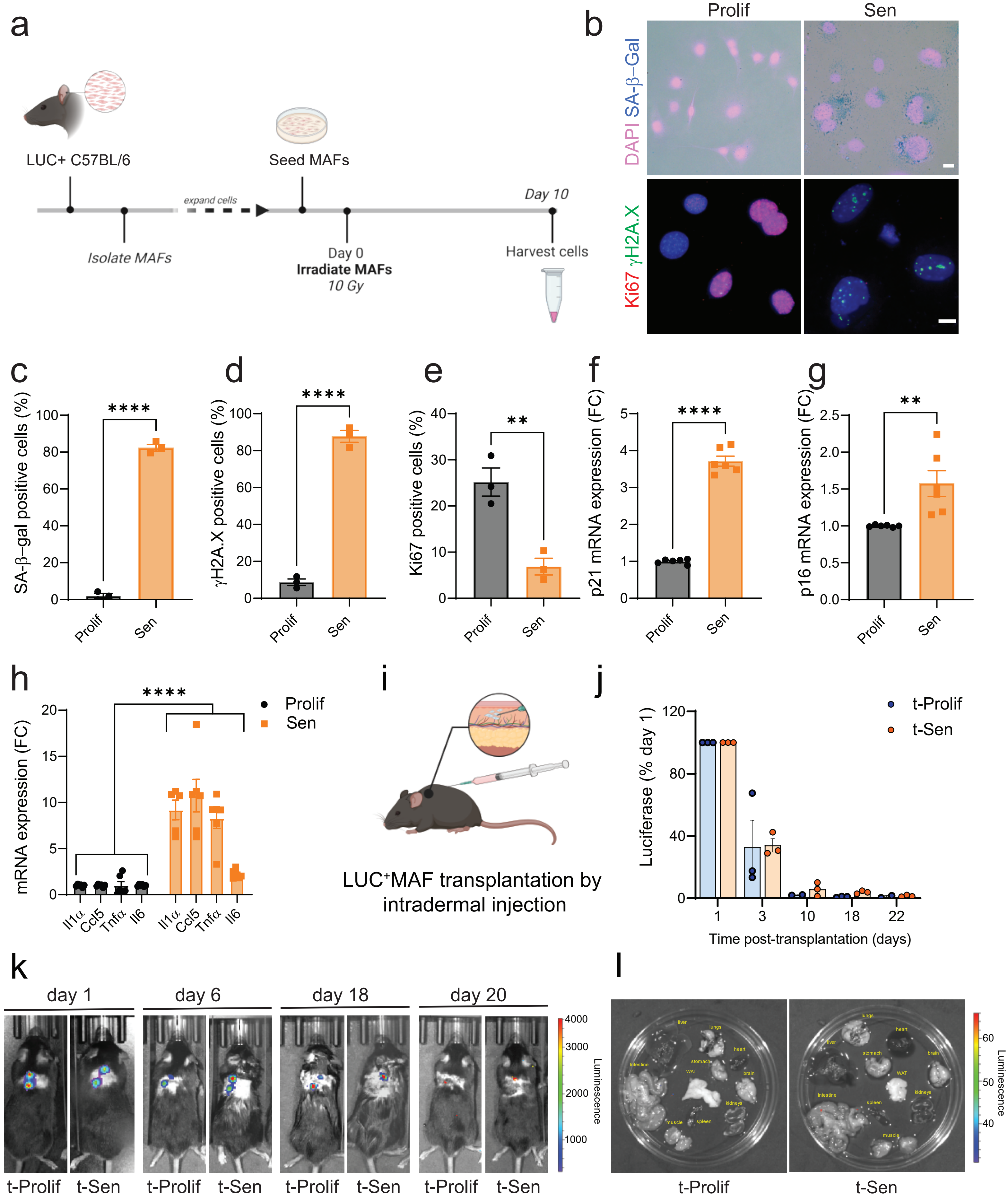
a
LUC+ C57BL/6
Isolate MAFs
expand cells
Seed MAFs
Day 0
Irradiate MAFs
10 Gy
Day 10
Harvest cells
b
Prolif
Sen
DAPI SA-β-Gal
Ki67 γH2A.X
c
SA-β-gal positive cells (%)
****
Prolif
Sen
d
γH2A.X positive cells (%)
****
e
Ki67 positive cells (%)
**
f
p21 mRNA expression (FC)
****
g
p16 mRNA expression (FC)
**
h
mRNA expression (FC)
****
Prolif
Sen
Il1α
Ccl5
Tnfα
Il6
i
LUC⁺MAF transplantation by intradermal injection
j
Luciferase (% day 1)
t-Prolif
t-Sen
Time post-transplantation (days)
k
day 1
day 6
day 18
day 20
t-Prolif
t-Sen
Luminescence
l
t-Prolif
t-Sen
Luminescence

Supplementary Figure 1 Transplantation of senescent in mouse ear fibroblasts into young mice skin.

a) scheme depicting isolation and induction of senescence in mouse adult fibroblasts (MAFs) isolated from ears of LUC+ mice; **b)** Representative images of SA-β-Gal (above) and Ki67/γH2A.X dual immunofluorescence (scale bar 10μm). Quantification of **c)** SA-b-Gal positive cells; **d)** γH2A.X positive cells; **e)** Ki67 positive cells and mRNA expression by q-PCR of **f)** p21, **g)** p16 and **h)** SASP factors Il1α, Ccl5, Tnfα and Il6 in proliferating (Prolif) and Senescent (Sen) mouse adult fibroblasts. **i)** 3 months old C57BL/6 male mice were transplanted with and $1x10^6$ senescent or proliferative MAFs in 2 sites of the dorsal skin's dermal layer; **j)** Quantification of the luminescence of mice at different times after transplantation (n=3) **k)** Presence of both transplanted proliferating (t-Prolif) and senescent (t-Sen) fibroblasts was monitored using IVIS Lumina In Vivo Imaging System. **l)** Representative IVIS images of different organs collected 2 months after transplantation. Data are mean±S.E.M of n=3-5. $^*p < 0.05$, $^{**}p < 0.01$ and $^{****}p < 0.0001$

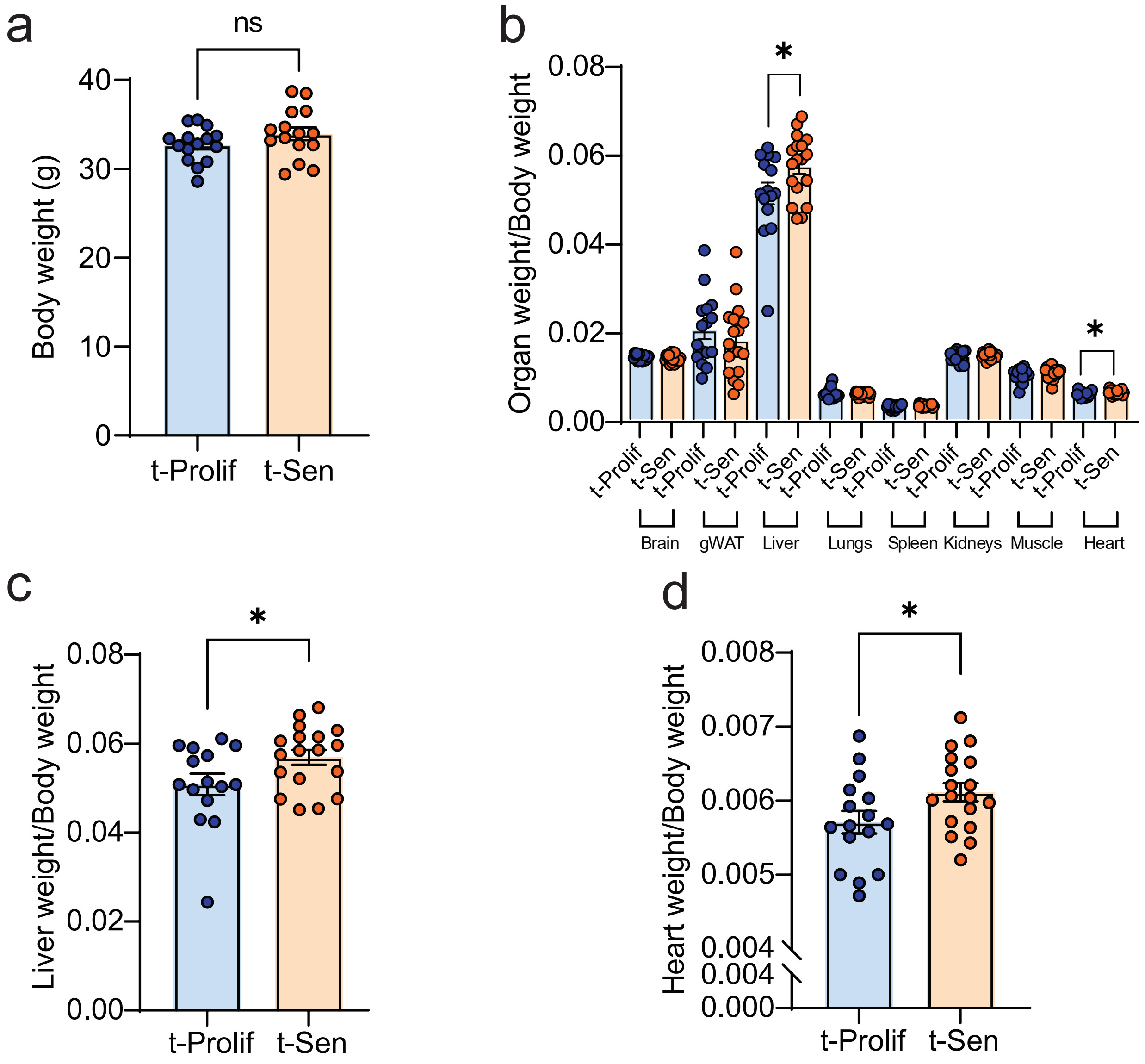

a
ns
Body weight (g)
40
30
20
10
0
t-Prolif
t-Sen
b
Organ weight/Body weight
0.08
0.06
0.04
0.02
0.00
*
*
t-Prolif
t-Sen
Brain
gWAT
Liver
Lungs
Spleen
Kidneys
Muscle
Heart
c
*
Liver weight/Body weight
0.08
0.06
0.04
0.02
0.00
t-Prolif
t-Sen
d
*
Heart weight/Body weight
0.008
0.007
0.006
0.005
0.004
0.004
0.000
t-Prolif
t-Sen

Supplementary Figure 2 Intradermal transplantation of senescent cells does not impact on body weight **a**) or the weight of different host organs **b**) but significantly increased liver **b** & **c**) and heart weights **b** & **d**) when normalized by body weight. Data are mean±S.E.M of n=15-18 animals per group. *$p < 0.05$.

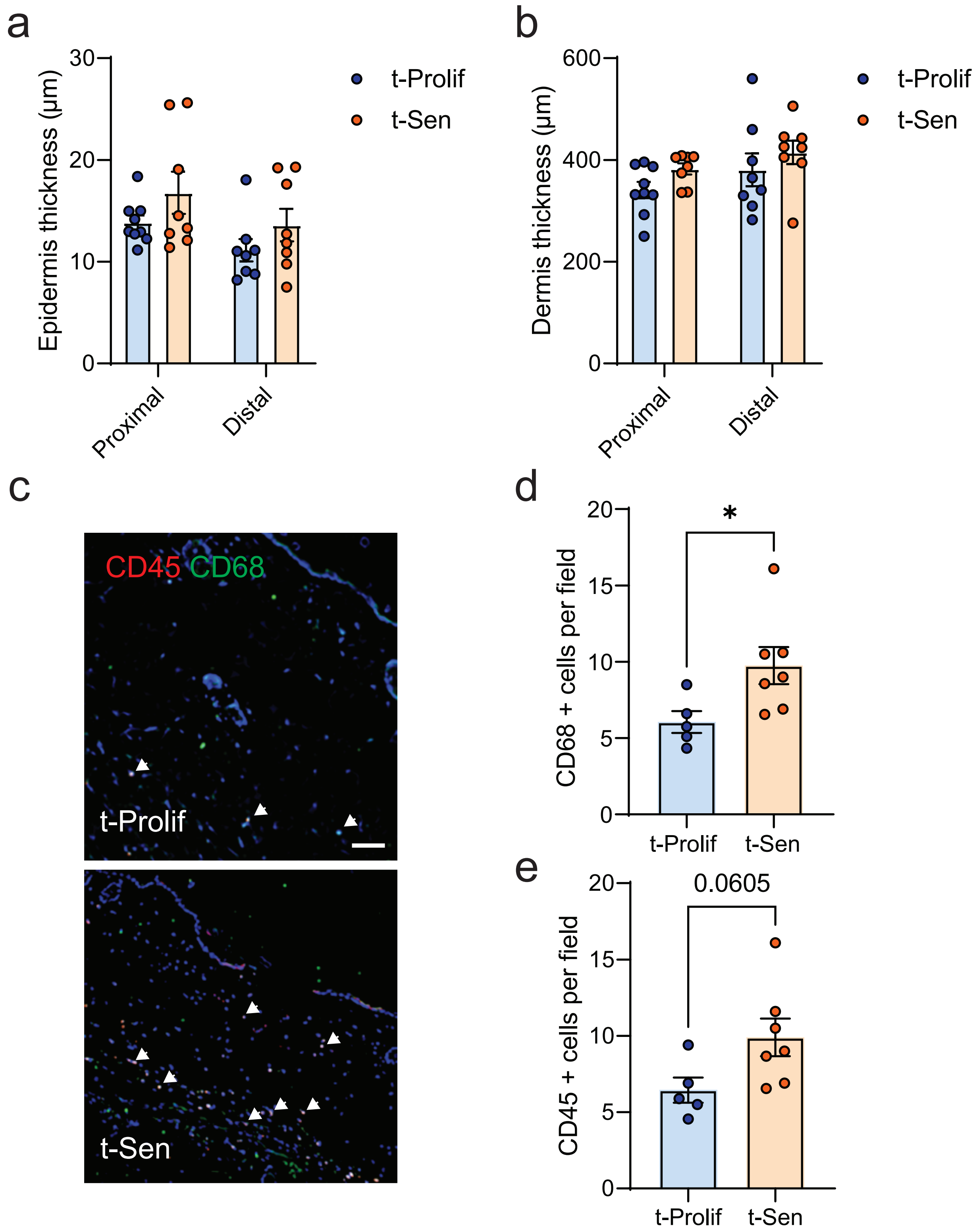

a
Epidermis thickness (μm)
0
10
20
30
Proximal
Distal
t-Prolif
t-Sen
b
Dermis thickness (μm)
0
200
400
600
Proximal
Distal
t-Prolif
t-Sen
c
CD45 CD68
t-Prolif
t-Sen
d
CD68 + cells per field
0
5
10
15
20
*
t-Prolif
t-Sen
e
CD45 + cells per field
0
5
10
15
20
0.0605
t-Prolif
t-Sen

Supplementary Figure 3

Intradermal transplantation of senescent cells does not impact on **a)** epidermal thickness and **b)** dermal thickness regardless of distance to injection site. **c)** Representative microscopy images of CD45 (in red) and CD68 (in green) (Scale bar 50µm) Quantification of average number of **d)** CD68 and **e)** CD45 positive cells per field. Data are mean±S.E.M of n=5-8 animals per group.

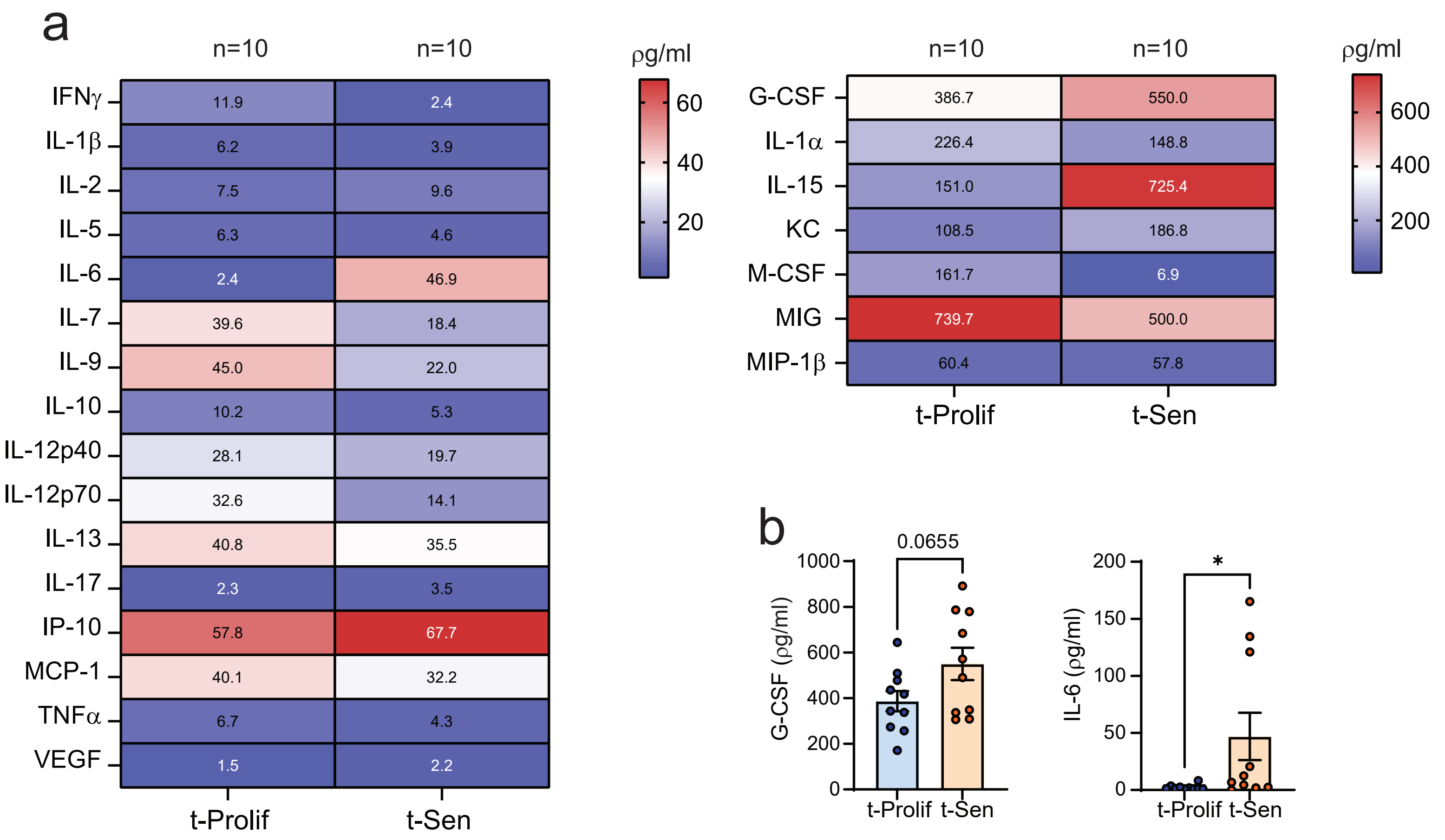
a
n=10
n=10
IFNγ
IL-1β
IL-2
IL-5
IL-6
IL-7
IL-9
IL-10
IL-12p40
IL-12p70
IL-13
IL-17
IP-10
MCP-1
TNFα
VEGF
11.9
2.4
6.2
3.9
7.5
9.6
6.3
4.6
2.4
46.9
39.6
18.4
45.0
22.0
10.2
5.3
28.1
19.7
32.6
14.1
40.8
35.5
2.3
3.5
57.8
67.7
40.1
32.2
6.7
4.3
1.5
2.2
t-Prolif
t-Sen
ρg/ml
60
40
20
n=10
n=10
G-CSF
IL-1α
IL-15
KC
M-CSF
MIG
MIP-1β
386.7
550.0
226.4
148.8
151.0
725.4
108.5
186.8
161.7
6.9
739.7
500.0
60.4
57.8
t-Prolif
t-Sen
ρg/ml
600
400
200
b
G-CSF (ρg/ml)
0.0655
1000
800
600
400
200
0
t-Prolif
t-Sen
IL-6 (ρg/ml)
*
200
150
100
50
0
t-Prolif
t-Sen

Supplementary Figure 4 Effect of senescent cell transplantation on the expression of inflammatory molecules in plasma

a) Heat map of different cytokines expression in the plasma of t-Prolif or t-Sen mice 5 months after transplantation. IL-6 **b)** and G-CSF **c)** were increased in t-Sen mice compared to t-Prolif controls. Data are mean±S.E.M of n=10 animals per group.

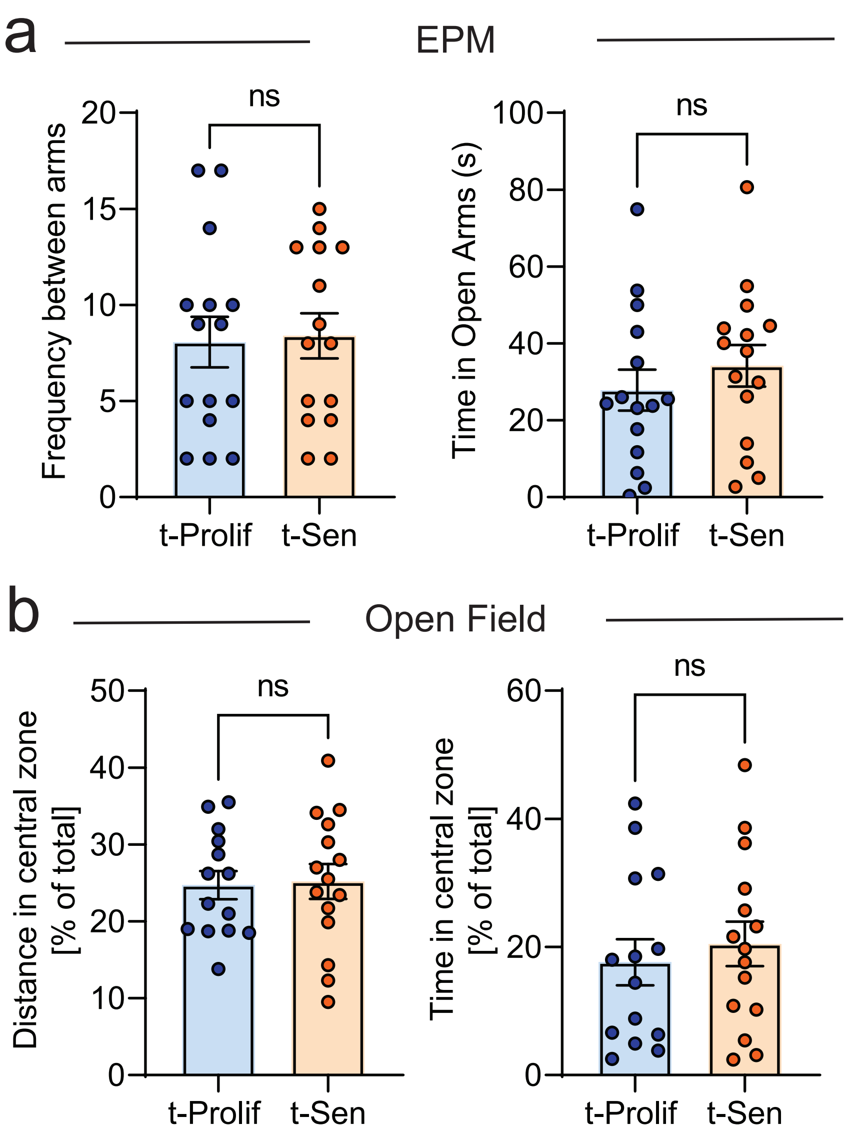

a
EPM
ns
Frequency between arms
20
15
10
5
0
t-Prolif
t-Sen
ns
Time in Open Arms (s)
100
80
60
40
20
0
t-Prolif
t-Sen
b
Open Field
ns
Distance in central zone
[% of total]
50
40
30
20
10
0
t-Prolif
t-Sen
ns
Time in central zone
[% of total]
60
40
20
0
t-Prolif
t-Sen

Supplementary Figure 5 Intradermal transplantation of senescent cells does not impact on anxiety-like behavior using the Elevated Plus Maze (EPM) and Open Field tests.

Effects of intradermal transplantation of proliferating (t-Prolif) and Senescent (t-Sen) fibroblasts on **a)** (left) frequency between arms and (right) time in open arms using EPM and **(b)** (left) distance in central zone and (right) time in central zone using the Open Field test. Data are mean±S.E.M of n=15 animals per group.

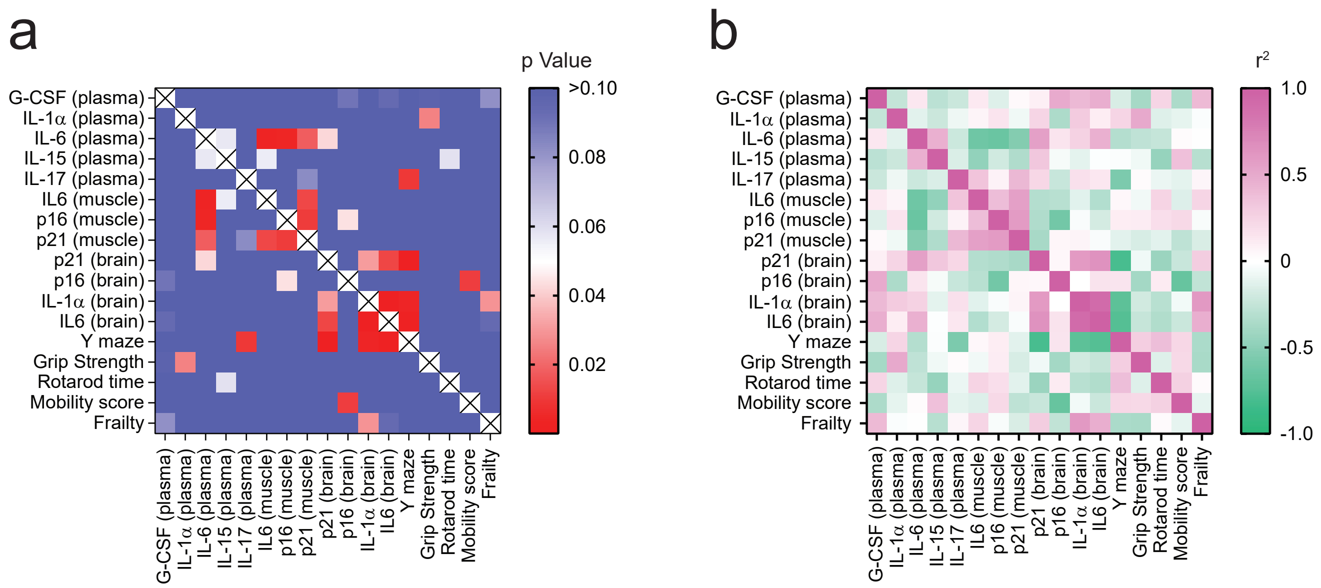

a
p Value
>0.10
0.08
0.06
0.04
0.02
G-CSF (plasma)
IL-1α (plasma)
IL-6 (plasma)
IL-15 (plasma)
IL-17 (plasma)
IL6 (muscle)
p16 (muscle)
p21 (muscle)
p21 (brain)
p16 (brain)
IL-1α (brain)
IL6 (brain)
Y maze
Grip Strength
Rotarod time
Mobility score
Frailty
b
r2
1.0
0.5
0
-0.5
-1.0

Supplementary Figure 6 Correlation matrix of senescence-associated markers and phenotypic assessments in mice transplanted with t-Prolif and t-Sen cells.

The left heat map displays p-values, indicating the statistical significance of each correlation, while the right heat map shows R-squared values, representing the strength and direction of the correlations.
